# Hepatocellular carcinoma with regional lymphadenopathy caused by sarcoid-like reaction: a case report

**DOI:** 10.1186/s40792-021-01146-2

**Published:** 2021-03-04

**Authors:** Tomoko Mizota, Masato Suzuoki, Saya Kaku, Kenichi Mizunuma, Kazuto Ohtaka, Ryo Takahashi, Kazuteru Komuro, Nozomu Iwashiro, Masanori Ohara, Noriko Kimura, Satoshi Hirano

**Affiliations:** 1 Department of Surgery, National Hospital Organization Hakodate National Hospital, 18-16, Kawahara-cho, Hakodate, Hokkaido 041-8512 Japan; 2 Department of Pathology, National Hospital Organization Hakodate National Hospital, 18-16, Kawahara-cho, Hakodate, Hokkaido 041-8512 Japan; 3 Department of Gastroenterological Surgery II, Hokkaido University Graduate School of Medicine, Kita 15 Nishi 7, Kita-ku, Sapporo, Hokkaido 060-8638 Japan

**Keywords:** Sarcoid-like reaction, Sarcoidosis, Granuloma, Epithelioid cells, HCC, Neoplasms, Lymphadenopathy

## Abstract

**Background:**

Sarcoid-like reaction (SLR) is a histological pattern of granulomatous inflammation that is clinically differentiated from sarcoidosis. Since SLR is known to occur in several neoplasias and occasionally causes lymphadenopathy and mimics metastatic malignancy, it needs to be considered whether lymphadenopathy is due to metastasis or SLR for the choice of cancer treatment. Few cases of hepatocellular carcinoma (HCC) with SLR have been reported. Here, a case of HCC with lymphadenopathy diagnosed as SLR without metastasis is presented.

**Case presentation:**

A 69-year-old woman was admitted to our hospital because of upper abdominal pain. She tested positive for hepatitis C virus ribonucleic acid. Imaging modalities showed an 81 × 65-mm-sized tumor with multiple nodules in segment 3 and a 17 × 12-mm-sized tumor in segment 5 with a common HCC enhancement pattern. In addition, a lymph node in the hepatoduodenal ligament was enlarged at 13 mm in size, suggesting the metastasis of HCC. Hepatectomy of the lateral segment and segment 5 and lymph node dissection in the hepatoduodenal ligament were performed. Both tumors in segments 3 and 5 were pathologically diagnosed as HCC without vessel invasion. The tumors contained necrotic cells and epithelioid cell granulomas with multinucleated giant cells, which is typically observed in sarcoidosis. The dissected lymph nodes also contained epithelioid cell granulomas, as well as giant cells with asteroid bodies. There was no malignancy in the lymph nodes. The pathological findings suggested the coexistence of malignancy and sarcoidosis. However, since the patient did not show any typical findings of pulmonary or cardiac sarcoidosis, the case was diagnosed as HCC with SLR in the primary lesion and regional lymph nodes.

**Conclusions:**

SLR needs to be considered in the differential diagnosis when a cancer patient develops lymphadenopathy. However, lymphadenopathy due to SLR is indistinguishable from that due to metastasis even when using multiple imaging modalities. Pathological examinations may be helpful for the diagnosis.

## Background

Sarcoid-like reaction (SLR) is a histological pattern of granulomatous inflammation related to a variety of pathophysiological conditions, such as infection, drugs, autoimmunity and neoplasms, and SLR is clinically differentiated from sarcoidosis with malignancy [1–4]. SLR has been reported to occur in 4–14% of malignancies, such as malignant lymphoma and carcinomas of the skin, breast, and lung [1]. SLR does not show any symptoms or fulfill diagnostic criteria for systemic sarcoidosis; therefore, specific treatment of sarcoidosis is not required. However, SLR occasionally causes lymphadenopathy and mimics metastatic malignancy. When regional lymph nodes near a malignancy become enlarged, it needs to be considered whether this enlargement is due to metastasis or SLR for the decision of cancer treatment. Although SLR is known to occur in several neoplasias, few cases of hepatocellular carcinoma (HCC) with SLR have been reported. The present case involved HCC with lymphadenopathy caused by SLR without any metastases.

## Case presentation

**Fig. 1:**
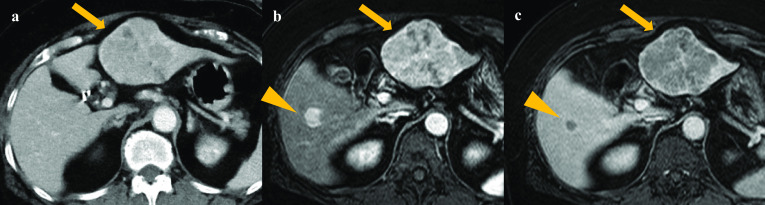
Preoperative imaging of the liver tumors. The enhanced CT scan showed an 81 × 63-mm-sized tumor in segment 3 (**a**) (arrow). The rim of the tumor was enhanced, and the inside of nodules showed hypoattenuation. Another 17 × 12-mm-sized tumor was found in segment 5 (**b**, **c**) (triangle) in addition to the liver in segment 3 (arrow) on Gd-EOB-DTPA-MRI. Both tumors were highly enhanced in the early arterial phase (**b**), followed by washout in the delayed phase (**c**). *CT* computed tomography; *Gd-EOB-DTPA* gadolinium ethoxybenzyl diethylenetriamine pentaacetic acid; *MRI* magnetic resonance imaging

A 69-year-old woman with a past history of hypertension was admitted to our hospital because of upper abdominal pain. She tested positive for hepatitis C virus (HCV) ribonucleic acid (RNA) for the first time, though she had not been given a transfusion. Laboratory data on admission showed a slightly high inflammatory response with a white cell blood count of 5200/mm^3^ and C-reactive protein level of 0.64 mg/dL. Aspartate transaminase was elevated at 42 IU/L, and alanine transaminase was within a normal range of 27 IU/L. Other liver enzymes were also elevated as the following: alkaline phosphatase of 479 IU/mL, leucine aminopeptidase of 93 IU/L, and gamma-glutamyl transpeptidase of 55 IU/L. Serum albumin was within a normal range of 3.9 g/dL. The tumor marker test revealed a high protein induced by vitamin K absence or antagonist-II (PIVKA-II) level of 181 mAU/ml and a normal alpha-fetoprotein (AFP) of 7 ng/ml. The enhanced computed tomography (CT) scan showed an 81 × 65-mm-sized tumor with multiple nodules in segment 3 of the liver (Fig. 1a).

The rim of the tumor was enhanced, and the inside of nodules showed hypoattenuation. Through gadolinium ethoxybenzyl diethylenetriamine pentaacetic acid (Gd-EOB-DTPA)-enhanced magnetic resonance imaging (MRI), a 17 × 12-mm-sized tumor was found in segment 5 in addition to the tumor in segment 3 (Fig. 1b, c). Both tumors were highly enhanced in the early arterial phase, followed by washout in the delayed phase, which is a common enhancement pattern of HCC.

**Fig. 2:**
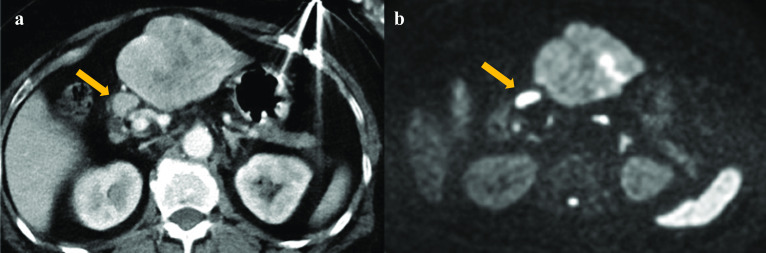
Lymphadenopathy on enhanced CT and diffusion-weighted MRI. A regional lymph node in the hepatoduodenal ligament was enlarged at 13 mm in size (**a**). The lymph node showed high signal on diffusion-weighted magnetic as well as liver tumors (**b**). It was suspected to be metastasis. *CT* computed tomography; *MRI* magnetic resonance imaging

In addition, a lymph node in the hepatoduodenal ligament was enlarged at 13 mm in size (Fig. 2a). The enhancement pattern of the lymph node was similar to liver tumors on Gd-EOB-DTPA-MRI. Furthermore, both two liver tumors and the lymph node showed high signal on diffusion-weighted MRI, which suggested lymph node metastasis of HCC (Fig. 2b). No other suspicious lesions, such as mediastinal or hilar lymph node enlargement, were found. Although she was an HCV carrier, her Child–Pugh score was A. Despite the enlarged lymph node suggesting metastasis, all lesions seemed to be resectable. Therefore, hepatectomy of the lateral segment, partial segment 5 liver resection and enlarged lymph node dissection in the hepatoduodenal ligament were performed. Since the enlarged lymph node in the hepatoduodenal ligament was found to adhere to the lymph nodes around the common hepatic artery during the operation, they were resected all together.

**Fig. 3:**
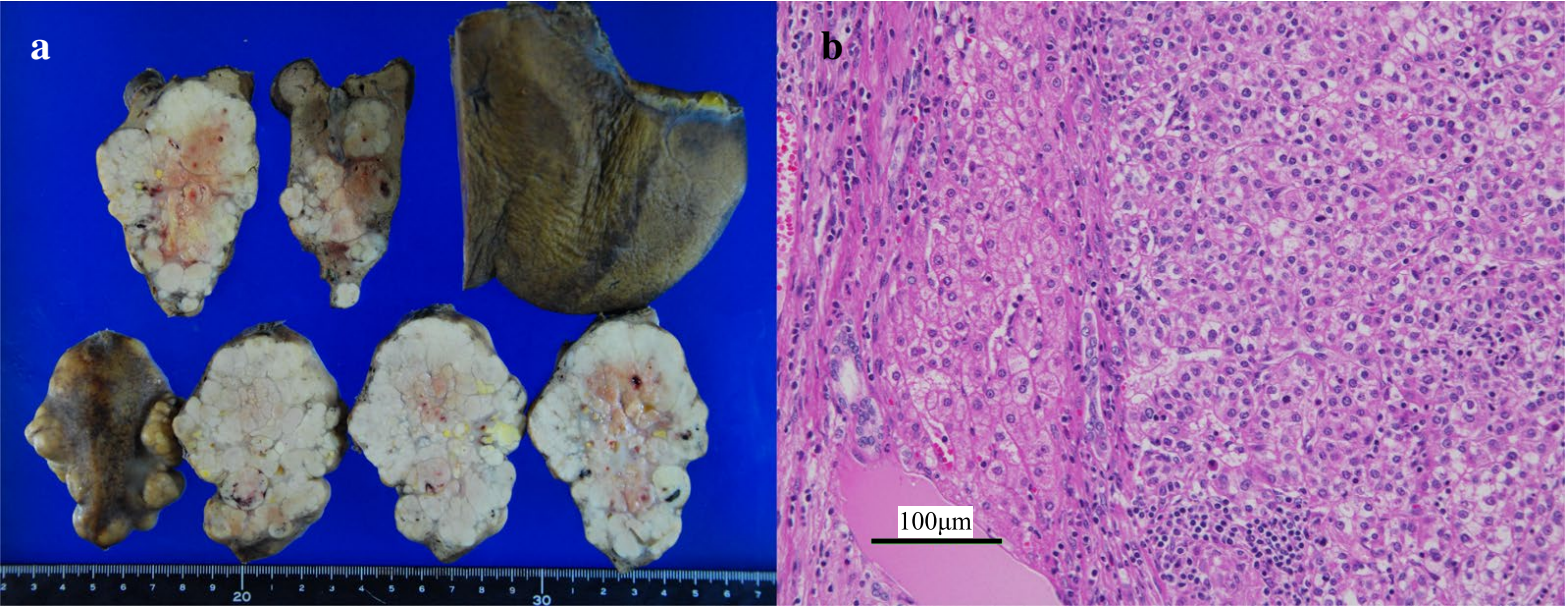
Pathological findings of the liver tumors. Both tumors comprised multiple nodules (**a**), and hematoxylin and eosin staining showed high-density tumor cells ranging from well to moderately differentiated hepatocellular carcinoma (**b**)

**Fig. 4:**
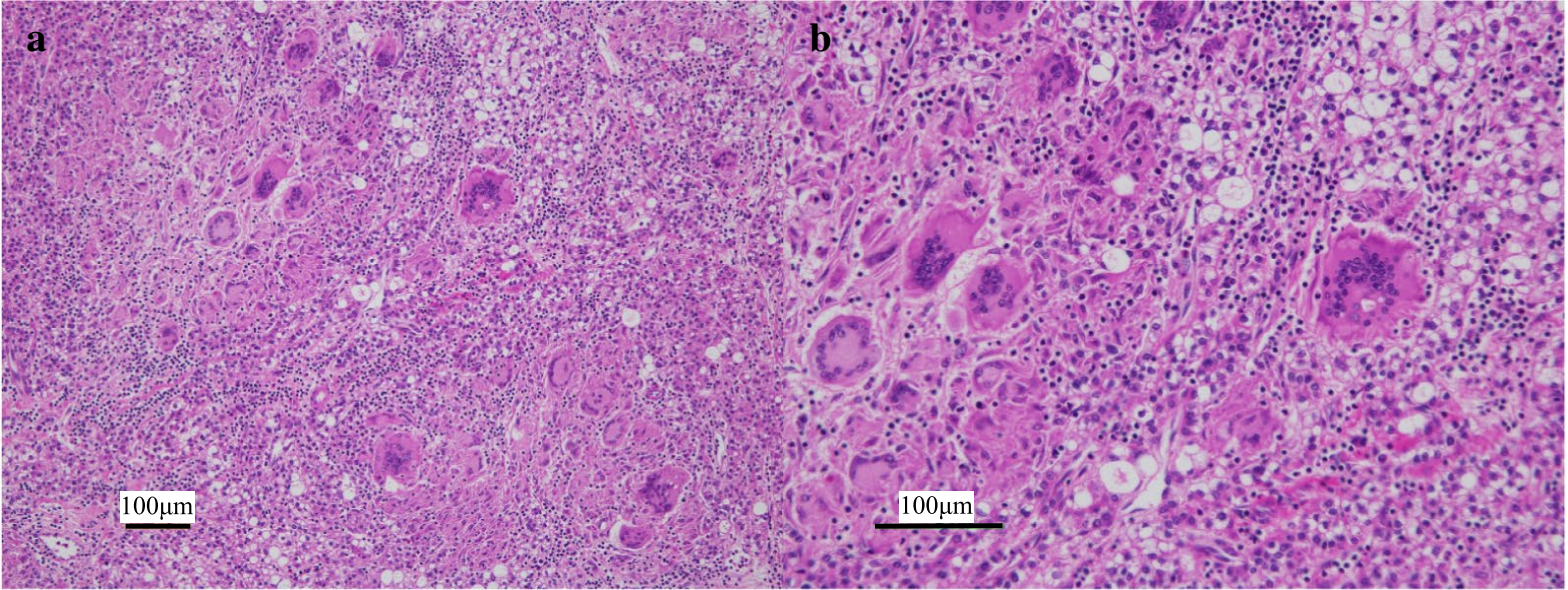
Pathological findings of sarcoid-like reaction in the liver. Epithelioid cell granulomas with multinucleated giant cells were included in the tumors (**a**, **b**)

Both tumors in segments 3 and 5 were white and solid and comprised multiple nodules (Fig. 3a). Hematoxylin and eosin (HE) staining showed high-density tumor cells ranging from well to moderately differentiated HCC with a large nucleus–cytoplasm ratio without vessel invasion (Fig. 3b). The tumors contained necrotic cells and epithelioid cell granulomas with multinucleated giant cells, which is typically observed in sarcoidosis (Fig. 4a, b). The liver tissue showed pathologically moderate chronic hepatitis, but not cirrhosis.

**Fig. 5:**
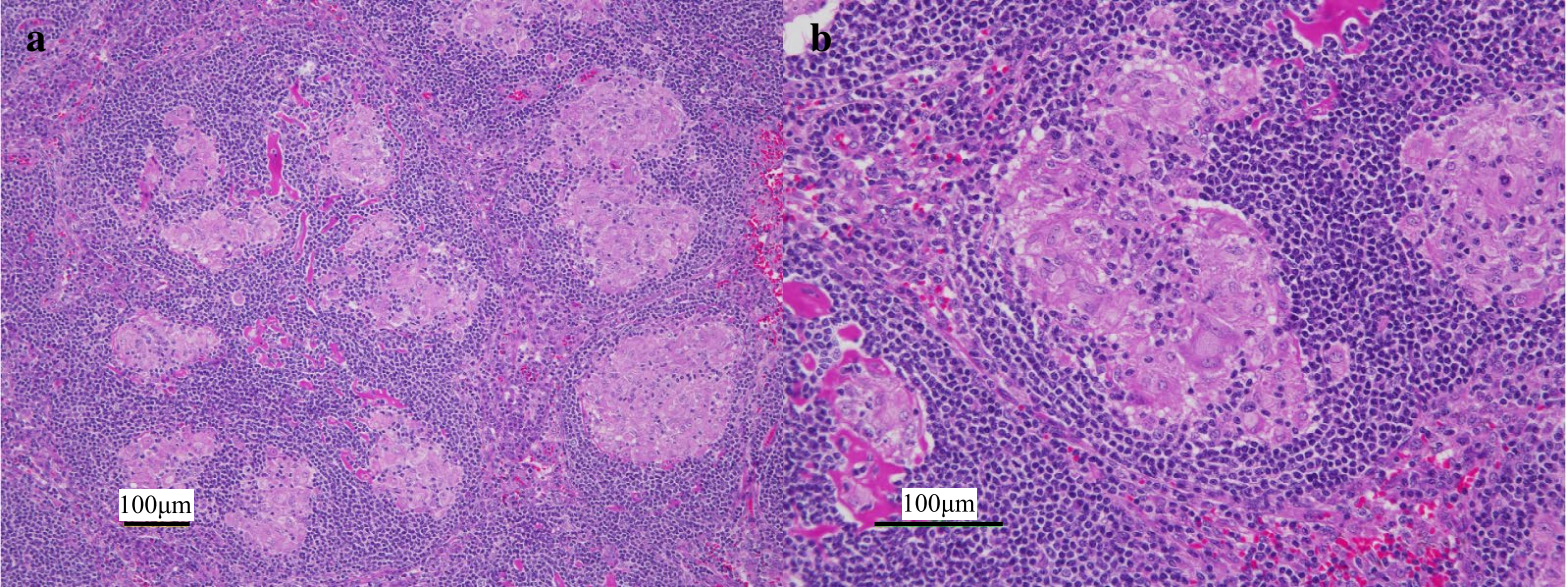
Pathological findings of the regional lymph nodes. The regional lymph nodes contained epithelioid cell granulomas without malignant findings (**a**, **b**)

Macroscopic findings of the dissected lymph nodes were also white and solid. They contained epithelioid cell granulomas, as well as giant cells with asteroid bodies (Fig. 5a, b). There was no malignancy in the dissected lymph nodes. The pathological findings suggested the coexistence of malignancy and sarcoidosis. However, since the postoperative angiotensin converting enzyme (ACE) level was within the normal range of 11.2 IU/L or any typical findings of pulmonary or cardiac sarcoidosis, the case was diagnosed as HCC with SLR in the regional lymph node.

Thirteen months after the surgery, multiple recurrent tumors in the liver were surgically resected. Lymph nodes that remained in hepatoduodenal ligament were found enlarged during the operation, and they were also dissected. All of these specimens including lymph nodes were pathologically diagnosed as metastasis surrounded by SLR. Although lenvatinib mesylate was orally administered, she died 17 months after the first surgery due to additional tumor recurrence in the liver.

## Discussion

In the present case, regional lymph nodes near the HCC were enlarged by SLR, but not by metastasis. The enlarged lymph nodes were preoperatively considered metastasis, but the pathological diagnosis of the surgical specimen was granulomas without malignancy. In addition, this case did not show any other lymphadenopathy in the mediastinum, the hilum of the lung or the abdominal cavity, which supported the diagnosis of SLR.

At present, only three studies of HCC with SLR have been reported, searching PubMed from 1966 to 2020 using the following terms: “hepatocellular carcinoma” [Title/Abstract] AND (“sarcoid reaction” OR “sarcoid-like reaction” OR “granuloma” OR “epithelioid”)[Title/Abstract]) (Table 1) [5–7]. In these three cases, SLR was found in a primary lesion of the liver. Our patient was the first reported case of HCC with SLR not only in the original tumor, but also in regional lymph nodes. When lymphadenopathy is found in a case of malignancy, it needs to be considered whether it is caused by metastasis or SLR because it can affect the choice of a treatment plan for malignancy. In our case, although lymphadenopathy was preoperatively suspected to be metastasis, surgical resection was performed because R0 resection seemed possible. Therefore, regardless of metastasis or SLR, surgical resection would have been performed for the patient. However, once SLR is preoperatively considered unresectable in the case of multiple enlarged lymph nodes, for instance, the opportunity for surgical treatment can be missed, although it is actually resectable. Gebrekidan et al. [8] presented the case of a patient with Hodgkin’s disease with SLR who was primarily diagnosed with Hodgkin’s disease having multiple enlarged lymph nodes in the iliac and inguinal regions and received chemotherapy. After chemotherapy, newly enlarged mediastinal and hilar lymph nodes were found and pathologically diagnosed as SLR following biopsy. Even during the treatment of malignancy, when inconsistent lymphadenopathy is detected, the possibility of SLR needs to be considered to avoid misdiagnosis or inappropriate management.

**Table 1:** Previously reported HCC cases with SLR, including the present case

Author	Published	Sex	Age	Specimen with SLR	Treatment	Survival
Tomimatsu et al. [5]	1982	Male	55	Liver	Intra-arterial injection chemotherapy	25 days
Schmidt et al. [6]	1985	Female	14	Liver	Operation	2 years alive
Mourra et al. [7]	2001	Male	54	Liver	Transplanted (without preoperative diagnosis as malignancy)	Not mentioned
Present case	2021	Female	69	Liver and lymph nodes	Hepatectomy, lymphadenectomy	17 months

Only three cases of hepatocellular carcinoma with SRL have been reported prior to the present case. SRL was found only in the primary lesion in the three previous cases, while our case had findings both in the primary lesion and regional lymph nodes

*HCC* hepatocellular carcinoma, *SLR* sarcoid-like reaction

However, lymphadenopathy due to SLR is preoperatively indistinguishable from that due to metastasis using multiple imaging modalities, such as ultrasound, CT, or MRI. In our case, the enlarged lymph node dissected during the first operation was suspected metastasis, but did not pathologically include malignant findings. On the other hand, while lymphadenopathy was not preoperatively detected on recurrence, pathological diagnosis was lymph node metastasis. The lymph node metastasis might have resulted from cancer growth of intrahepatic recurrence. However, preoperative diagnosis of the lymph node metastasis was not possible. In recent decades, the use of ^18^F-fluorodeoxyglucose (FDG)-positron emission tomography (PET)/CT has increased for cancer follow-up. Koo et al. [9] reviewed patients who underwent transbronchial lymph node aspiration biopsy for mediastinal and hilar lymph nodes and investigated the FDG uptake level of lymphadenopathies of SLR and malignant lymphoma. Multivariate analysis showed that the total volume of lymph nodes was significantly smaller, and the number of enlarged lymph nodes was larger in SLR than in malignant lymphadenopathy. On the other hand, there was no significant difference in maxSUV between these two types of lymphadenopathy. Since hypermetabolic uptake can be observed in both benign and malignant lesions, they noted that ^18^F-FDG-PET/CT findings were not helpful. Another publication also reviewed some cases of SLR in ^18^F-FDG-PET/CT and reported that SLR and metastasis were indistinguishable [10]. So far, no imaging modality has enabled us to differentiate lymph nodes with SLR from those with malignancy. If possible, pathological examinations, such as biopsy including preoperative endoscopic ultrasound-fine needle aspiration (EUS-FNA) and intraoperative frozen section, may be useful for the diagnosis.

Previously reported cases of SLR had bilateral enlargement of mediastinal and/or hilar lymph nodes, and FDG uptake was detected [11]. Such cases can be more easily suspected not only for metastasis, but also for SLR. On the other hand, we are not aware of the identification of SLR using preoperative imaging modalities in a case such as ours with only regional lymphadenopathy. The present patient did not undergo ^18^F-FDG-PET/CT imaging. Even if it had been performed, since no other lymphadenopathy was found in the abdominal cavity, mediastinum or hilum of the lung, FDG uptake might have not been detected other than in the regional lymph node. SLR always needs to be considered a differential diagnosis when a cancer patient develops lymphadenopathy.

Previous publications indicated that malignancy with SLR had a better prognosis than malignancy without SLR in patients with Hodgkin’s disease and gastric cancer [12, 13]. Sacks et al. [12] reviewed 608 patients with Hodgkin’s disease and noted that patients with granulomas had a longer relapse-free survival period and more prolonged overall survival than those without granulomas. Another lung cancer case was reported in which SLR disappeared with the growth of cancer, which suggested that cancer growth might have impaired immunity [14]. It was hypothesized that the SLR was related to an antineoplastic immune response [10]. Soluble antigens from neoplasia enhance immunity, forming epithelioid cell granulomas. This theory suggested that the enhanced immunity of patients with SLR of malignancy might result in a better prognosis than in those without SLR. It is unknown whether these findings and hypotheses can be generalized in every SLR that occurs in malignancy. With regard to HCC with SLR, since only a few cases have been reported, its prognosis has not been clarified. More cases are needed for additional investigation.

## Conclusion

Hepatectomy and lymph node dissection were performed on HCC with regional lymphadenopathy, which was suspected metastasis but pathologically diagnosed as SLR. SLR needs to be considered as a differential diagnosis when a cancer patient develops lymphadenopathy. Lymphadenopathy with SLR is indistinguishable from that with metastasis using imaging modalities. Pathological examinations may be helpful for the diagnosis.

## Data Availability

The data supporting the conclusions of this case report are included within the article.

## Abbreviations

SLR: Sarcoid-like reaction
HCC: Hepatocellular carcinoma
HCV: Hepatitis C virus
RNA: Ribonucleic acid
PIVKA-II: Protein induced by vitamin K absence or antagonist-II
AFP: Alpha-fetoprotein
ACE: Angiotensin converting enzyme
CT: Computed tomography
Gd-EOB-DTPA: Gadolinium ethoxybenzyl diethylenetriamine pentaacetic acid
MRI: Magnetic resonance imaging
HE: Hematoxylin and eosin
FDG: ^18^F-fluorodeoxyglucose
PET: Positron emission tomography
EUS-FNA: Endoscopic ultrasound-fine needle aspiration
